# Supplementary material for: Prudent-Active and Fast-Food-Sedentary Dietary-Lifestyle Patterns: The Association with Adiposity, Nutrition Knowledge and Sociodemographic Factors in Polish Teenagers—The ABC of Healthy Eating Project
Source: Nutrients. 2018 Dec 15;10(12):1988. doi: 10.3390/nu10121988 (PMC6315952; doi:10.3390/nu10121988)
Supplement: Supplementary file 1 [file nutrients-10-01988-s001.pdf]

nutrients

MDPI

Supplementary material: Prudent-Active and Fast-food-Sedentary dietary-lifestyle patterns: the association with adiposity, nutrition knowledge and sociodemographic factors in Polish teenagers. The ABC of Healthy Eating Project.

Lidia Wadolowska, Jadwiga Hamulka, Joanna Kowalkowska, Małgorzata Kostecka, Katarzyna Wadolowska, Renata Biezanowska-Kopec, Ewa Czarniecka-Skubina, Witold Kozirok, Anna Piotrowska

Table S1. Standardized means of components of dietary-lifestyle patterns identified with cluster analysis.

Variables	Prudent-Active	Fast-Food-Sedentary	notPrudent-notFast-Food-LowActive	p-Value
Sample size	454	214	881	
Consumption of				
Breakfast [a]	**0.20**	−0.48	0.01	****
School meal [b]	**0.37**	−0.47	−0.08	****
Frequency consumption of [c]				
Vegetables	**0.85**	−0.34	−0.36	****
Fruits	**0.84**	−0.31	−0.35	****
Fruit or vegetable juices	**0.72**	0.00	−0.37	****
Dairy products	**0.51**	0.04	−0.28	****
Fish	**0.35**	0.08	−0.20	****
Fast-foods	−0.21	**1.32**	−0.21	****
Sweetened beverages	−0.10	**1.29**	−0.27	****
Energy drinks	−0.23	**1.15**	−0.16	****
Sweets	−0.04	**1.03**	−0.24	****
Screen time [d]	−0.30	**1.10**	−0.11	****
Physical activity at school [e]	**0.37**	−0.18	−0.14	****
Physical activity at leisure time [f]	**0.43**	−0.36	−0.14	****

Before standardization the input variables were expressed in: [a] times/day after converting frequencies (into daily frequency): <1 day/week (0), 1 to 3 days/week (0.29), 4 to 6 days/week (0.71), every day (1); [b] times/day after converting frequencies (into daily frequency): <1 day/week (0), 1 to 3 days/week (0.29), 4 to 6 days/week (0.71), every school day (1); [c] times/day after converting frequencies (into daily frequency): never or almost never (0), rarely than once a week (0.06), once a week (0.14), 2–4 times/week (0.43), 5-6 times/week (0.79), every day (1), several times a day (2); [d] scores after converting categories (into scores): <2 h/day (1), 2 to <4 hours/day (2), 4 to <6 h/day (3), 6 to <8 h/day (4), 8 to <10 h/day (5), ≥10 h/day (6); [e] scores after converting categories (into scores): low, *e.g.* most of the time in a sitting position, in class or on breaks (1), moderate, *e.g.* half the time in a sitting position and half the time in motion (2), vigorous, *e.g.* most of the time on the move or on classes related to high physical exertion (3); [f] scores after converting categories (into scores): low, *e.g.* more time spent sitting, watching TV, in front of a computer, reading, light housework, a short walk to 2 h a week (1), moderate, *e.g.* walking, cycling, gymnastics, working at home or other light physical activity performed 2–3 h/week (2), vigorous, *e.g.* cycling, running, working at home or other sports activities requiring physical effort over 3 h/week (3). Food examples by groups: Vegetables, *e.g.* fresh, boiled, baked, stewed; Fruits, *e.g.* fresh or frozen; Dairy products, *e.g.* milk, yoghurt, cottage cheese, cheese; Fish, *e.g.* baked, fried, in vinegar, canned; Fast foods, *e.g.* chips, pizza, hamburgers; Sweetened drinks, *e.g.* cola type, water with syrup, tea type with sugar; Sweets, *e.g.* cookies, sweets, cake, bars of chocolate, chocolate. Statistically significant: **** $p < 0.0001$.
